# Predictive value of spot versus 24-hour measures of proteinuria for death, end-stage kidney disease or chronic kidney disease progression

**DOI:** 10.1186/s12882-018-0853-1

**Published:** 2018-03-07

**Authors:** Tracey Ying, Philip Clayton, Chetana Naresh, Steven Chadban

**Affiliations:** 1 Level 6 Renal Department, Royal Prince Alfred Hospital, Missenden Rd, Camperdown, Australia; 2 Sydney Medical School, University of Sydney, Camperdown, Australia; 3 Central Northern Adelaide Renal and Transplantation Service, Royal Adelaide Hospital, Adelaide, Australia; 4 Australia and New Zealand Dialysis and Transplant (ANZDATA) Registry, Adelaide, Australia; 5 School of Medicine, University of Adelaide, Adelaide, Australia; 6 The Cairns Hospital, Cairns, Australia

**Keywords:** Protein-to-creatinine ratio, Albumin-to-creatinine ratio, Proteinuria, Albuminuria, Chronic kidney disease, Renal outcomes

## Abstract

**Background:**

Proteinuria is well recognised as a marker of chronic kidney disease (CKD), as a risk factor for progression of CKD among those with known CKD, and as a risk factor for cardiovascular events and death among both the general and CKD populations. Which measure of proteinuria is most predictive of renal events remains uncertain.

**Methods:**

We conducted a prospective study with 144 proteinuric CKD and kidney transplant recipients attending an outpatient clinic of a tertiary care hospital in Australia. We concurrently collected morning spot urine protein-to-creatinine ratio (UPCR), albumin-to-creatinine ratio (UACR) and 24-h urinary protein excretion (24-UPE) from each participant at baseline. The primary outcome was a composite of death, ESKD or > 30% decline in eGFR over 5-years. Secondary outcomes were each component of the composite outcome. For each proteinuria measure, we performed a Cox Proportional Hazards model and calculated the Harrell’s C-statistic and Akaike’s Information Criterion (AIC).

**Results:**

After a mean follow-up of 5 years (range 4.4–6), 85 (59%) patients met the primary composite outcome including 23 deaths (16%). The multivariable analysis showed strong evidence of an association between each log-transformed proteinuria measurement and the primary composite outcome. [Log-UPCR 1.31 (95% CI 1.18–1.63), log-UACR 1.27 (1.11–1.45) and log-24-UPE 1.43 (1.20–1.71)]. The C-Statistic were similar for all three measures of proteinuria [UPCR: 0.74 (95% CI: 0.69–0.80), UACR: 0.75 (0.69–0.81), 24-UPE: 0.75 (0.69–0.81)] as were the models’ AIC (671, 668 and 665 respectively). For secondary outcomes, no proteinuria measure was significantly associated with death alone ([log-UPCR = 1.18 (0.96–1.84), log-UACR = 1.19 (1.00–1.55), log-24-UPE = 1.19 (0.83–1.71)], whilst UACR and 24-UPE demonstrated marginally better association with ESKD and > 30% decline in eGFR respectively. [For ESKD, adj log-UACR HR = 1.33 (1.07–1.66). For > 30% decline in eGFR, log-24-UPE adj HR = 1.54 (1.13–2.09)].

**Conclusion:**

In patients with stable, non-nephrotic CKD, all three measures of proteinuria were similarly predictive of hard clinical endpoints, defined as a composite of death, ESKD and > 30% decline in eGFR. However, which measure best predicted the outcomes individually is less certain.

## Background

Proteinuria is an established marker of kidney damage, a risk factor for progression of chronic kidney disease (CKD) and a potent risk factor for cardiovascular events and mortality amongst the diabetic and non-diabetic population [1, 2]. Precise measurements of proteinuria allow the clinician to identify patients at risk of CKD progression and to monitor response to treatment. Major clinical practice guidelines now recommend using a spot urine albumin-to-creatinine ratio (UACR) as first-line in the evaluation of proteinuria for the diagnosis of CKD and monitoring response to treatment [3–6]. Previously considered the gold standard, there has been an increasing movement to forgo the timed 24-h protein excretion as the test of choice to quantify proteinuria due to the inconvenience and inaccuracies associated with the test [7, 8]. In contrast, studies of patients with glomerular diseases have shown only modest correlations between UPCR compared to 24-h protein excretion [9, 10] however; these studies did not evaluate long-term outcomes. Underpinning such guidelines, studies of morning spot UACR have shown to be the superior method for predicting renal events compared with 24-h protein and albumin excretion in patients with diabetic nephropathy [11]. Therefore, which measure of proteinuria remains most predictive of renal events remains a key question and has not been definitively answered. We sought to determine the relationship between the three most common lab-based measurements of proteinuria (spot UPCR, UACR and 24-h urinary total protein excretion) and clinical outcomes over five years in a cohort of CKD patients at a tertiary care hospital. We hypothesised that UPCR and UACR are non-inferior to 24-h total protein excretion in predicting clinical progression and outcomes for patients with CKD.

## Methods

### Study design, setting and participants

We conducted this single-centre prospective longitudinal study at a metropolitan tertiary care teaching hospital in Sydney, Australia. At baseline in 2008–2010, we studied 270 CKD and kidney transplant patients collecting spot UACR, UPCR and 24-h urinary protein excretion as previously described [12, 13]. In summary, medically stable CKD patients were instructed at baseline to perform a 24-h urine collection and to collect multiple spot untimed urine samples over the 24-h period. We obtained the baseline UPCR and UACR from the morning (not first void) samples. The fresh specimens were returned to the hospital at the end of the 24-h collection period and analysed in the hospital’s centralised laboratory within 48 h. At the time of the urine collection, participants also underwent a blood test to determine haemoglobin, urea and serum creatinine. eGFR was derived using the isotope dilution mass spectrometry (IDMS) traceable Chronic Kidney Disease Epidemiology Collaboration (CKD-EPI) formula. Patients were followed up in the clinic at an interval determined by their caring physician. We followed this cohort of patients for five years to determine their clinical status. We only included patients in the study if the results for all three baseline measures of proteinuria were available and the patient had at least one follow-up visit.

### Variables

We obtained the following baseline variables at recruitment: age, sex, ethnicity, cause of CKD, eGFR, serum creatinine, kidney transplant status, angiotensin converter enzyme inhibitor or angiotensin receptor blocker use, history of diabetes mellitus (DM) (defined as a previous diagnosis of DM, use of oral hypoglycaemic agent or insulin), hypertension (defined by a history of diagnosed hypertension or the use of antihypertensive medications) and smoking history. The predictor variables were baseline UPCR, UACR and 24-h urinary protein excretion. The primary outcome was a composite of all-cause death, ESKD requiring dialysis or preemptive kidney transplantation or > 30% decline in eGFR. We chose a composite outcome as the primary outcome, after balancing the consideration of statistical precision, with the clinical significance of the composite outcome [14]. Secondary outcomes included the individual components of all-cause death, progression to renal replacement therapy (RRT) and a greater than 30% decline in eGFR.

The first author and data assistant manually searched the electronic medical record database of the local area health district, supplemented by the Australian and New Zealand Dialysis and Transplant Registry (ANZDATA) to obtain dates of death, dialysis commencement and patient disposition at five years. If a patient was alive and had not reached ESKD, we recorded their serum creatinine and eGFR within 3 months of the 5-year follow-up date. The Sydney Local Area Health district ethics committee approved the study (protocol no. X16–0269).

### Statistical methods

For descriptive statistics, we reported normally distributed continuous variables as means (standard deviation [SD]) and skewed continuous variables as medians (interquartile range [IQR]). For categorical data, we reported absolute numbers and percentages. We performed a Cox proportional hazards model for the primary composite and individual secondary outcomes. Because the data for the predictor variables UPCR, UACR and 24-h total protein excretion were highly skewed, we performed log-transformations on these variables. We excluded cases with missing predictor or outcome variables in the analysis.

To decide which measure of proteinuria is most predictive of the primary composite outcome, we constructed a Cox regression model and calculated the Harrell’s C-statistic and Akaike’s Information Criterion (AIC) for each measure of proteinuria. The C-statistic is a measure of predictive power (where 0.5 = random concordance and 1 = perfect concordance) and the AIC is a measure of the model’s goodness-of-fit. For the Cox model, we first performed a univariable analysis using the predictor variables. For the multivariable models, we added variables that were known risk factors for progression of CKD including age, baseline eGFR, hypertension and diabetes mellitus and any predictor variable with a *p*-value of less than 0.1 in the univariable analysis. To check whether the association between the predictor variables and the primary outcome was modified by whether the patient had a functioning transplant at the time of study enrolment, we created an interaction term between each of the predictor variables and the transplant status. We checked the proportional hazard assumption of each model by plotting the Schoenfield residuals against time. We considered a two-tailed *P* value of < 0.05 as statistically significant. We performed a sensitivity analysis by excluding patients who had a functioning kidney transplant at study recruitment. We assessed the linearity of each predictor variable using Martingale Residuals and by plotting the residuals against the predictor variable using LOWESS (locally weighted scatterplot smoothing). We analysed the data using Stata, version 14.1 (StataCorp LP, College Station TX).

## Results

### Participants

**Fig. 1:**
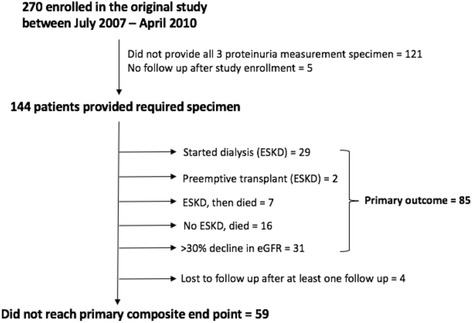
Study participant and flow

Figure 1 provides the study participant flow. Of 270 patients consented and enrolled at baseline, 144 patients provided all three measurements of proteinuria and had follow-up data available and were included in the present study.

### Descriptive data

Table 1 shows the baseline characteristics of the study participants. There was minimal missing data (one each for the cause of CKD, smoking status and DM type). The mean age was 54 years, and 58.4% were males. The predominant cause of CKD was glomerulonephritis (37.5%), followed by hypertension (21.5%) and diabetic nephropathy (11.8%). 42 (29.2%) patients had a functioning kidney transplant at the time of study recruitment. The main ethnicities were Caucasians (*n* = 81, 56.3%), followed by Mediterranean (*n* = 35, 24.3%) and Asian (*n* = 22, 15.3%). The median baseline eGFR estimated by the CKD-EPI equation was 44 ml/min/1.73m^2^ (IQR 28–65.5 ml/min/1.73m^2^). 50 (34.7%) of the patients had microalbuminuria (3–30 mg/mmol), whilst the majority of the patient [79 (54.9%)] had macroalbuminuria (> 30 mg/mmol) [3]. 18 (12.5%) patients had nephrotic-range proteinuria defined as more than 3 g of protein excretion over 24-h. The median protein excretion as measured by UPCR, UACR and 24-h protein excretion were 0.06 g/mmol (IQR 0.02–0.16), 41.9 mg/mmol (IQR 9.4–130.8) and 0.6 g/day (IQR 0.2–1.7) respectively.

**Table 1:** Baseline characteristics of study participants

Variable	Value
Age at baseline	54 years (SD 15.6)
Male	86 (58.4%)
Ethnicity	
Caucasian	81 (56.3%)
Mediterranean	35 (24.3%)
Asian	15 (10.4%)
Indian/subcontinent	7 (4.9%)
Other	6 (4.1%)
Primary disease	
GN	54 (37.5%)
DM	17 (11.8%)
HTN	31 (21.5%)
PCKD	11 (7.6%)
Other	30 (20.9%)
Missing	1 (0.7%)
Baseline creatinine (μmol/L)	137 (IQR 98–205)
Baseline eGFR (ml/min/1.73m^2^)	44 (IQR 28–65.5)
UPCR (g/mmol)	0.06 (IQR 0.02–0.16)
UACR (mg/mmol)	41.9 (IQR 9.4–130.8)
24 h protein excretion (g/day)	0.6 (IQR 0.2–1.7)
HTN	
Yes	113 (78.5%)
On ACE-I	
Yes	119 (82.6%)
Smoking status	
Never	71 (49.3%)
Former	61 (42.4%)
Current	11 (7.6%)
Unknown	1 (0.7%)
DM	
Yes	50 (34.7%)
Type 1	3 (6%)
Type 2	46 (90%)
Missing	1 (2%)
Functioning Transplant	
Yes	42 (29.2%)

Data given as means (Standard deviation [SD]) or median (interquartile range [IQR])

*GN* glomerulonephritis, *DM* diabetes mellitus, *HTN* hypertension, *PCKD* polycystic kidney disease, *eGFR* estimated glomerular filtration rate, *UPCR* urine protein-to-creatinine ratio, *UACR* urine albumin-to-creatinine ratio, *ACE-I* angiotensin converting enzyme inhibitor, *DM* diabetes mellitus

### Outcome data

We followed patients for a mean duration of 5 years (range 4.4–6 years). There were 85 (59%) participants that met the primary composite outcome, including 23 patients (16%) who died during the follow-up period (Fig. 1).

### Correlation of UPCR, UACR and 24-h protein excretion with death, ESKD or > 30% decline in eGFR (primary composite outcome)

**Fig. 2:**
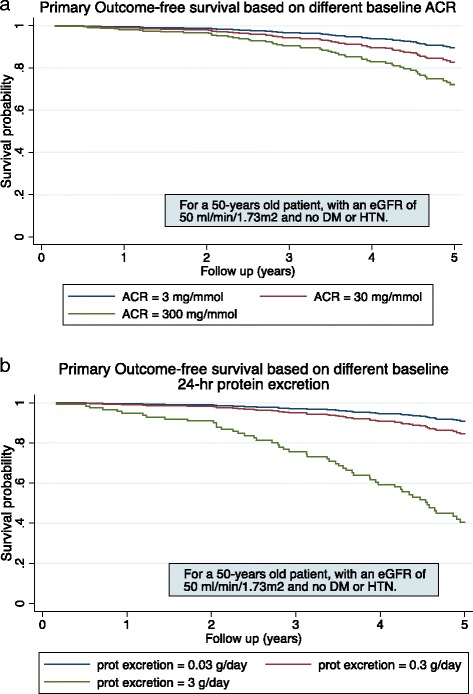
Kaplan-Meier survival curves for the primary outcome for a hypothetical patient as measured by (**a**) albumin-to-creatinine ratio and (**b**) 24-h urine protein excretion. Note: survival probability = survival free from death, end-stage kidney disease or > 30% decline in estimated glomerular filtrate rate ACR = albumin-to-creatinine ratio, DM = diabetes mellitus, HTN = hypertension, prot = protein

Univariable analysis of log UPCR, UACR and 24-h protein excretion showed very strong evidence of association with the primary composite outcome [Log-UPCR hazard ratio (HR) = 1.39, 95% CI 1.18–1.62, *p* <  0.001. Log-UACR HR = 1.29, 95% CI 1.13–1.47, *p* <  0.001. Log-24-h protein excretion = 1.43, 95% CI 1.21–1.68, *p* <  0.001). When adjusted for age, eGFR, hypertension and diabetes mellitus status, the association remained highly significant [Log-UPCR adjusted (adj) HR = 1.31, 95% CI 1.18–1.63, *p* = 0.001. Log-UACR adj HR = 1.27, 95% CI 1.11–1.23, *p* <  0.001. Log-24-h protein excretion adj HR = 1.43, 95% CI 1.20–1.78, *p* <  0.001)]. The models are summarised in Table 2. To interpret these results graphically, Fig. 2a and b show the probability of renal survival in a hypothetical 50-year patient with an eGFR of 50 ml/min/1.73m^2^ and no history of hypertension or diabetes mellitus. With increasing baseline albuminuria and proteinuria, there is a separation of curves in both measurements of proteinuria.

**Table 2:** Cox Proportional Hazards Model for the risk of death, ESKD or > 30% decline in eGFR (primary composite outcome)

	Univariable model			Multivariable model		
	Log PCR	Log ACR	Log 24-h protein excretion	Log PCR	Log ACR	Log 24-h protein excretion
HR (95% CI)	1.39 (1.18–1.62)	1.29 (1.13–1.47)	1.43 (1.21–1.68)	1.31 (1.18–1.63)	1.27 (1.11–1.23)	1.43 (1.20–1.71)
*P*- value	< 0.001	< 0.001	< 0.001	0.001	< 0.001	< 0.001
Harrell’s C statistic (95% CI)	0.64 (0.57–0.70)	0.64 (0.57–0.71)	0.64 (0.57–0.71)	0.74 (0.69–0.80)	0.75 (0.69–0.81)	0.75 (0.69–0.81)
AIC	691	691	689	671	668	665

The multivariable model is adjusted for baseline age, eGFR, hypertension and diabetes mellitus

*ESKD* end-stage kidney disease, *eGFR* estimated glomerular filtration rate, *HR* hazard ratio, *CI* confidence interval, *AIC* Akaike information criterion, *UPCR* urine protein-to-creatinine ratio, *UACR* urine albumin-to-creatinine ratio

**Fig. 3:**
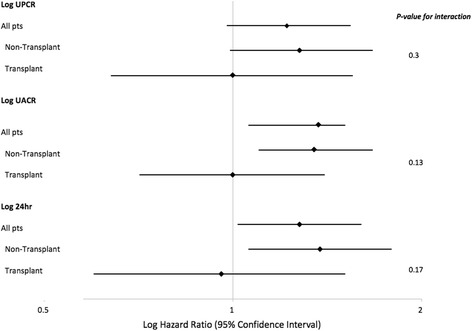
Forest plot showing the adjusted hazard ratio and 95% confidence interval of the association between proteinuria measures and the primary outcome. The association between the three baseline measures of proteinuria and the primary composite outcome shown for all patients, CKD (non-transplant) patients and transplant patients. The multivariable model for “all patients” is adjusted for baseline age, eGFR, hypertension and diabetes mellitus. The model stratified by transplant status includes age, eGFR, hypertension, diabetes mellitus and the interaction term between transplant status and the measure of proteinuria. The *p*- value for the interaction term is shown. The diamonds represent the HR and the horizontal bars the 95% CI. UPCR = urine protein-to-creatinine ratio, Pts = patients, UACR = urine albumin-to- creatinine ratio, 24 h = 24-h protein excretion, HR = Hazard ratio, CI = confidence intervals, eGFR = estimated glomerular filtration rate

To examine whether transplant status modified the association between proteinuria and the primary outcome, we fitted interaction terms between transplant status and each measure of proteinuria (Fig. 3). In these analyses, none of the measures of proteinuria were predictive of the primary outcome in transplant recipients. However, as none of the interaction terms were statistically significant, it was not retained in the final models.

### The predictive power of spot versus 24-h urine protein with the primary composite outcome

All three baseline measures of proteinuria showed a similar predictive power for the primary outcome in the multivariable analysis and for the model’s goodness-of-fit. [Harrell’s C statistic with 95% CI: UPCR = 0.74 (0.69–0.81), UACR - 0.75 (0.69–0.81), 24-h protein excretion = 0.75 (0.69–0.81) and AIC: UPCR = 671, UACR = 668 and 24-h protein excretion = 665]. These are summarised in Table 2.

### Correlation of UPCR, UACR and 24-h protein excretion with individual secondary outcomes

Table 3 summarises the risk of individual clinically significant endpoints per log-increment of proteinuria measurement. In the multivariable model, none of the proteinuria measurements were significantly associated with death [log-UPCR = 1.18 (0.96–1.84), *p* = 0.3, log-UACR = 1.19 (1.00–1.55), *p* = 0.2, log-24-h protein excretion = 1.19 (0.83–1.71), *p* = 0.3]. Of the three measurements, 24-h protein excretion showed the strongest evidence of association with > 30% decline in eGFR per log-increment [24-h protein excretion adj HR = 1.54 (1.13–2.09) *p* = 0.005]. Finally, all three measurements were strongly associated with ESKD in the univariable analysis, with UACR and 24-h protein excretion remaining significant in the multivariable model after adjusting for age, baseline eGFR, diabetes and hypertension. (The HRs are displayed in Table 3).

**Table 3:** Cox proportional hazards model for the association of baseline measures of proteinuria and individual (secondary) outcomes

	Univariable model			Multivariable model		
	Death	ESKD	> 30% decline in eGFR	Death	ESKD	> 30% decline in eGFR
Log UPCR	1.42 (1.05–1.92) *p* = 0.02	1.48 (1.18–1.88) *p* = 0.001	1.34(1.01–1.77) *p* = 0.04	1.18 (0.96–1.84) *p* = 0.3	1.27 (0.97–1.66) *p* = 0.08	1.29 (0.97–1.71) *p* = 0.09
Log UACR	1.26 (0.99–1.60) *p* = 0.06	1.42 (1.17–1.73) *p* < 0.001	1.36 (1.00–1.55) *p* = 0.06	1.19 (0.92–1.55) *p* = 0.2	1.33 (1.07–1.66) *p* = 0.009	1.21 (0.98–1.50) *p* = 0.08
Log 24-h protein excretion	1.28 (0.94–1.73) *p* = 0.1	1.47 (1.17–1.85) *p* = 0.001	1.62 (1.20–2.19) *p* = 0.002	1.19 (0.83–1.71) *p* = 0.3	1.39 (1.07–1.82) *p* = 0.02	1.54 (1.13–2.09) *p* = 0.005

The multivariable model was adjusted for age, baseline eGFR, diabetes mellitus and hypertension

*UPCR* urine protein-to-creatinine ratio, *UACR* urine albumin-to-creatinine ratio, *ESKD* end-stage kidney disease, *eGFR* estimated glomerular filtration rate

ESKD is defined as the requirement for dialysis or pre-emptive kidney transplantation

### Sensitivity analysis

We excluded participants who had a functioning kidney transplant at baseline and found similar adjusted HRs between baseline measures of proteinuria and the primary composite outcome [Log-UPCR adj HR = 1.41, (1.17–1.72), *p* = 0.001, log-UACR adj HR = 1.36, (1.16–1.60), *p* <  0.001 and log-24-h protein excretion adj HR = 1.43, (1.09–1.88), *p* <  0.001]. All baseline measures of proteinuria were also similarly predictive of the primary outcome (Harrell’s C statistic 0.77 vs. 0.78 vs 0.78 respectively) (Table 4).

**Table 4:** Multivariable Cox Proportional Hazards model for death, ESKD or > 30% decline in eGFR (primary composite outcome), excluding kidney transplant patients

	Log UPCR	Log UACR	Log 24-h protein excretion
HR (95% CI)	1.41 (1.17–1.72)	1.36 (1.16–1.60)	1.43 (1.09–1.88)
*P*- value	0.001	< 0.001	< 0.001
Harrell’s C statistic	0.77	0.78	0.78
AIC	429	412	410

The multivariable model was adjusted for age, baseline, diabetes mellitus and hypertension

*ESKD* end-stage kidney disease, *eGFR* estimated glomerular filtration rate, *HR* hazard ratio, *CI* confidence interval, *AIC* Akaike information criterion, *UPCR* urine protein-to-creatinine ratio, *UACR* urine albumin-to-creatinine ratio

## Discussion

In this single-centre study of 144 patients with CKD, our study showed that all three measures of proteinuria were strongly associated with the composite outcome of death, ESKD or > 30% decline in eGFR and were similarly predictive of clinically relevant renal outcomes as demonstrated by a similar Harrell’s C statistic [15].

Proponents of the untimed or spot proteinuria measurement method point out that UPCR is a reasonably accurate indicator of 24-h proteinuria and is a simple and less time-consuming way of measuring proteinuria excretion [16]. On the other hand, opponents have argued that the large diurnal and day-to-day variation in protein excretion make untimed spot testing an unreliable method of quantifying proteinuria [17]. More recently, this was shown in a study of 302 patients with glomerulonephritis, that reported only a modest correlation between UPCR and 24-h protein excretion [9]. Given these results, our study suggests that all three measures of proteinuria were similarly predictive of hard clinical outcomes, thus supporting the use of untimed spot collections over the more cumbersome timed collection.

Our study is unique due to the availability of both spot and 24-h proteinuria measures within the same individual. Only a handful of studies have directly compared all three measures of proteinuria in their ability to predict clinically significant events. These are summarised in Table 5. The largest study in a CKD population was a retrospective study of 5586 Scottish patients, of which 1676 had UPCR, UACR and 24-h measures (protein and albumin excretion) collected simultaneously [18]. The study showed similar adjusted HRs for death, renal replacement therapy and a doubling of SCr for all measurements, however, it is important to note that these ratios were derived from aliquots of a 24-h urine sample rather than spot samples. Two other studies comparing untimed and timed protein excretion have demonstrated that spot samples were equal or even superior to 24-h samples in predicting clinical outcomes. However, these studies were limited to IgA nephropathy [19] and diabetic nephropathy patients [11]. Finally, one other study of 98 proteinuric CKD patients evaluated UPCR versus 24-h urine protein excretion and found both measures to be equal in predicting progression of CKD. However, this study did not evaluate UACR. Another novel aspect of our study was the inclusion of transplanted patients amongst the CKD patients. The inclusion of patients with a functioning graft in our study is valid because multiple observational studies have shown a strong association between proteinuria and reduced graft survival [20–22]. Consistent with our findings, a large single-centre study has also shown that both spot and 24-h measures of albumin and protein excretion were similar predictors of doubling of serum creatinine, transplant loss and death.(18) The results of the current study are therefore in-line with previous studies in various CKD populations demonstrating that spot urine protein ratios provide valuable prognostic information.

**Table 5:** Studies comparing different measures of proteinuria in a CKD population in predicting death or progression of disease

Author	Study type	Population	Test	Outcome measure	Most predictive test
Zhao et al. 2016 [19]	Prospective cohort	438 Chinese patients with IgA nephropathy	UPCR UACR 24 h UPE	Composite of death, RRT or > 30% change in eGFR	UACR
Talreja et al. 2014 [25]	Prospective cohort	207 Canadian kidney transplant recipients	UPCR UACR 24 h UPE 24 h albumin excretion	Transplant loss, doubling of SCr or death	All tests similarly predictive
Methven et al. 2011 [18]	Retrospective cohort	1676 Scottish patients with CKD	UPCR UACR 24 h UPE 24 h-albumin excretion	All-cause death, RRT and doubling of SCr level	UPCR and UACR equal
Lambers Heerspink et al. 2010 [11]	Randomised controlled trial	701 patients with type 2 diabetes mellitus and CKD	UACR 24 h UPE 24 h albumin excretion	Doubling of SCr or ESKD	UACR
Ruggenenti et al. 1998 [26]	Cross sectional longitudinal	Subset study of 98 non-diabetic patients with CKD	UPCR 24 h UPE	eGFR decline Progression to ESKD	Both tests similarly predictive

*UPCR* urine protein-to-creatinine ratio, *UACR* urine albumin-to-creatinine ratio, *24 h UPE* 24-h urine protein excretion, *RRT* renal replacement therapy *eGFR* estimated glomerular filtration rate, *CKD* chronic kidney disease, *SCr* serum creatinine, *ESKD* end-stage kidney disease

Our study showed some mixed results on individual secondary outcomes, which should be interpreted in the context of a small number of events for each outcome. In the multivariable model, we did not detect a significant association between death and any of the proteinuria measures. For ESKD, we found a significant association with UACR and 24-protein excretion. In contrast, only 24-h urinary protein excretion was significantly associated with a >  30% decline in eGFR in the multivariable model. Spot UACR has been conclusively shown to be associated with both death and ESKD risk in the CKD prognosis consortium meta-analyses which included both the clinical and general population cohorts [1, 2, 23]. Our much smaller study confirmed a link with progression to ESKD for UACR and 24-h urinary protein excretion but no such relationship with mortality, indicating that the study was likely inadequately powered to detect an association.

Our study is limited by being a single-centre study with a relatively small number of patients, the majority being white males. Despite 270 patients initially enrolled, only 144 completed all three baseline proteinuria measurements. We measured eGFR change over 5-years, rather than 2-years, which is a generally more accepted surrogate endpoint [24]. The proportion of patients with nephrotic range proteinuria was small (12.5%), so our results should only be generalised to CKD patients with non-nephrotic range proteinuria. The strengths of the study are that this was a prospective cohort with very low numbers of loss to follow-up. There was a standardised urine collection procedure, and we only analysed fresh urine sample specimens. We captured all three commonly used measures of proteinuria for each participant and obtained objective and clinically relevant outcomes.

## Conclusion

In conclusion, our study supports the current clinical practice of collecting spot UACR or UPCR for prognostic information in patients with stable CKD with non-nephrotic range proteinuria. All three measures of proteinuria were similarly predictive of hard clinical outcomes over 5 years, defined as a composite of death, ESKD and > 30% decline in eGFR. The evidence is less certain when we consider each outcome individually. In a bid to assess the utility of UACR or UPCR as surrogate measures of hard outcomes, future research should focus on whether changes in the quantity of proteinuria over time in response to treatment or natural history, is predictive of a similar change in the risk of hard outcomes.

## Abbreviations

AIC: Akaike information criterion
CI: Confidence interval
CKD: Chronic kidney disease
CKD-EPI: Chronic kidney disease epidemiology collaboration formula
DM: Diabetes mellitus
eGFR: Estimated glomerular filtration rate
ESKD: End-stage kidney disease
HR: Hazard ratio
IQR: Interquartile range
RRT: Renal replacement therapy
SCr: Serum creatinine
SD: Standard deviation
UACR: Urine albumin-to-creatinine ratio
UPCR: Urine protein-to-creatinine ratio
UPE: Urinary protein excretion
